# Laparoscopic Cholecystectomy in Situs Inversus Totalis

**DOI:** 10.4103/1319-3767.37803

**Published:** 2008-01

**Authors:** Jamal Hamdi, Omar Abu Hamdan

**Affiliations:** Department of Surgery, Medical College, Umm Al-Qura University, Makkah, Saudi Arabia; * Hera General Hospital, Makkah, Saudi Arabia

**Keywords:** Laparoscopic cholecystectomy, situs inversus totalis

## Abstract

Situs inversus totalis is a rare defect with genetic predisposition that may present difficulties in the diagnosis and management of abdominal pathology due to mirror-image anatomy. Occasionally, these patients may present with acute cholecystitis. Laparoscopic cholecystectomy is the standard treatment for symptomatic cholelithiasis; however, the technique has to be varied for the treatment of situs inversus totalis. To the best of our knowledge, we report the first case in Saudi Arabia of a successful laparoscopic cholecystectomy in a patient with situs inversus totalis. The technique is presented and the pitfalls are discussed with a review of the relevant literature.

Situs inversus totalis is a rare congenital disorder occurring in 0.01% of the population.[1] It is characterized by the transposition of the major thoracic organs and all the visceral organs of the abdomen to the side opposite to normal position in the body. The liver and gall bladder are located on the left, while the stomach and the spleen are on the right. The normal development requires a 270 degree counterclockwise rotation that yields the normal anatomy. In situs inversus totalis, the 270 degree rotation is in the clockwise direction.[2] The exact etiology is unclear; however, it is thought to be due to a single autosomal recessive gene of incomplete penetration. The male to female ratio is 1:1 and there is no racial predilection.

In acute abdomen, it is important to be aware of the presence of situs inversus to ensure the correct diagnosis and treatment. Acute appendicitis causes left lower quadrant pain, whereas cholecystitis causes left upper quadrant pain in these patients. CT scanning is the preferred diagnostic modality as it shows the anatomical details.

Symptomatic cholelithiasis is very common in Saudi Arabia; however, it is extremely rare to find it in a patient with situs inversus due to the rarity of the latter conditions. We report the first case of successful laparoscopic cholecystectomy in a patient with situs inversus totalis in Saudi Arabia.

## CASE REPORT

**Figure 1:**
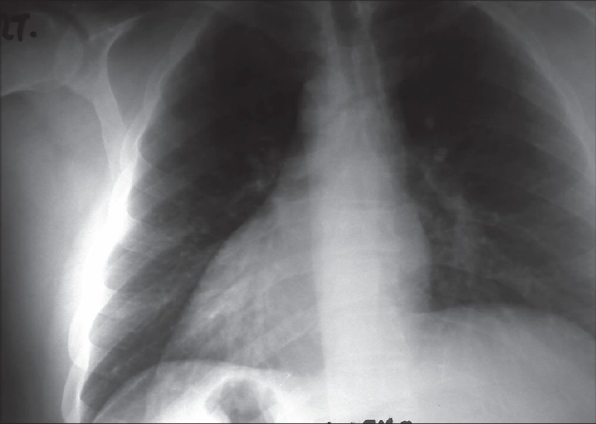
Chest X-ray showing dextrocardia

**Figure 2:**
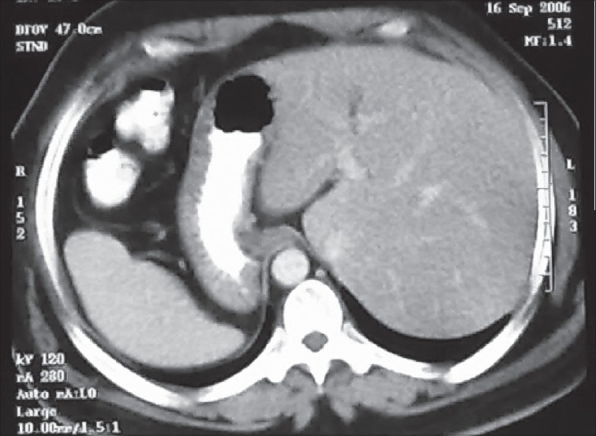
CT scan showing the transposition of major abdominal organs

A 41-year-old Saudi male with situs inversus totalis and peptic ulcer presented to the emergency room with 4-day history of pain in the left upper abdominal quadrant. The pain was colicky in nature, radiating to the back and this condition was aggravated by fatty meals. Physical examination showed positive Murphy's sign in the left hypochondrium Ultrasound showed the location of gallbladder on the left side of the body with a 13 mm stone impacted in its neck and signs of acute cholecystitis. Chest X-ray showed dextrocardia [Figure 1]. Laparoscopic cholecystectomy was performed on the following day. The patient made an uneventful recovery and was discharged home on the consecutive day. CT scan shows the transposition of the major abdominal organs [Figure 2].

### Technique

The patient was placed in the supine position with both the surgeon and camera-man on his right side and the assistant on the left side. There was one monitor that was placed near the head of the patient at the left side. A 5 mm laparoscope was introduced through an umbilical incision. A 10 mm trocar was introduced in the subxiphoid area in the mid-line, passing to the left side. Two 5 mm trocars were introduced in the left mid-clavicular and left anterior axillary lines. A grasper was introduced through the anterior axillary cannula to hold the fundus of the gallbladder and it was pushed laterally to the cephalic position. Another grasper was introduced through the medial cannula for the holding Hartmann's pouch and for manipulating it. This was initially held by the right hand of the surgeon. A dissector was introduced through the subxiphoid cannula and was manipulated by the left hand of the surgeon. This procedure proved to be difficult for a right-handed surgeon. The surgeon frequently changed his hands to grasp the dissector with his right hand, while the assistant held the Hartmann's pouch grasper or moved the dissector to the medial cannula and he used it with his right hand while holding the Hartmann's pouch grasper through the subxiphoid cannula with his left hand. Most of the Calot dissection as well as the application of the clips to both cystic artery and duct was performed through the subxiphoid port. The operation took nearly 2 h and was completed successfully.

## DISCUSSION

Situs inversus totalis is an extremely rare condition and performing successful laparoscopic cholecystectomy in these patients is even rarer. In July 2006, Bediou reported the 13^th^ case in the world. In the extensive search performed using MEDLINE, including the non-English language literature, only 20 cases were identified.[3] None of these cases were from Saudi Arabia. In our case, both the surgeons are right-handed and therefore the technique has to be adjusted. It is much easier for a left-handed surgeon to perform laparoscopic cholecystectomy in such patients.[4,5] Dissection from the mid-clavicular cannula with right hand with the lateral displacement of the neck of the gallbladder using the left hand through the subxiphoid cannula is difficult because the tip of the dissector will lose its perpendicular angle to the dissection plane and become positioned with a very narrow angle.[6] We performed the dissection alternatively from both cannulae. The dissection was quite safe and this confirms the previous reports of safe laparoscopic cholecystectomy in situs inversus totalis.[7,8]
